# Integrated Regulation of Immunity and Nutritional Symbiosis in Deep-Sea Mussels

**DOI:** 10.3390/md23110425

**Published:** 2025-10-31

**Authors:** Akihiro Tame

**Affiliations:** Faculty of Medical Sciences, Life Science Research Laboratory, University of Fukui, 23-3 Matsuoka Shimoaizuki, Eiheiji-cho, Yoshida-gun, Fukui 910-1193, Japan; atame@g.u-fukui.ac.jp; Tel.: +81-0776-61-8421

**Keywords:** *Bathymodiolus*, deep-sea mussel, intracellular symbiosis, innate immunity, phagocytosis, mTORC1, host-bacteria interaction, nutritional interaction, symbiosome

## Abstract

Deep-sea mussels of the genus *Bathymodiolus* exhibit adaptability to nutrient-poor deep-sea environments by establishing nutritional intracellular symbiosis with chemosynthetic bacteria harbored within the gill epithelial cells. However, this poses a conflict for the innate immune system of the host, which must balance the tolerance of beneficial symbiotic bacteria with the need to eliminate exogenous microbes. This review synthesizes existing knowledge and recent findings on *Bathymodiolus japonicus* to outline the cellular and molecular mechanisms governing this symbiotic relationship. In the host immune system, hemocytes are responsible for systemic defense, whereas gill cells are involved in local symbiotic acceptance. Central to the establishment of symbiosis is the host’s phagocytic system, which non-selectively engulfs bacteria but selectively retains symbionts. We highlight a series of cellular events in gill cells involving the engulfment, selection, retention and/or digestion of symbionts, and the regulatory mechanism of phagocytosis through mechanistic target of rapamycin complex 1, which connects bacterial nutrient supply with host immune and metabolic responses. This integrated model of symbiosis regulation, which links immunity, metabolism, and symbiosis, provides a fundamental framework for understanding how hosts establish and maintain a stable coexistence with microbes, offering a new perspective on symbiotic strategies in diverse organisms.

## 1. Introduction

Symbiotic relationships between eukaryotes and microorganisms have played a pivotal role in the evolution of life [1,2]. Nutritional symbiosis, in which microbial partners supply essential nutrients and metabolic cofactors to their host animals, is regarded as key to the survival and proliferation of eukaryotes in nutrient-poor environments [3,4,5,6]. From an evolutionary standpoint, nutritional symbioses have repeatedly emerged across animal lineages. Such partnerships, from corals and sponges to chemosynthetic bivalves, illustrate convergent strategies whereby immune and metabolic systems coevolve to accommodate beneficial microbes while restricting pathogens [7,8,9]. Such symbiotic strategies are also widely prevalent in organisms inhabiting deep-sea hydrothermal vents and cold seeps, where they enable animals to acquire nutrition independent of photosynthesis through associations with chemosynthetic bacteria [6,10,11].

A notable example of symbiosis is seen in the deep-sea mussels of the genus *Bathymodiolus*. These mussels harbor sulfur-oxidizing and methane-oxidizing bacteria within epithelial gill cells called bacteriocytes [6,12]. These symbionts perform carbon fixation using energy derived from the oxidation of inorganic reducing compounds, such as hydrogen sulfide and methane, while supplying organic nutrients to their hosts [6,13,14]. Host *Bathymodiolus* mussels exhibit a high dependence on nutrients obtained from their symbionts [6,14,15,16].

In most *Bathymodiolus* species, they acquire each symbiont from the environment through a “horizontal transmission” mode [17,18,19]. Each host mussel tends to selectively acquire a suitable symbiotic partner and maintain long-term symbiotic relationships through metabolic coordination [6,14,20,21]. However, this symbiotic relationship poses a conflicting demand for the evolutionarily conserved immune defense system in the host. Although the immune system recognizes and eliminates exogenous microbes, it must tolerate certain beneficial bacteria to maintain stable symbiotic relationships. These conflicting requirements raise a fundamental scientific question: “How do host mussels select symbionts from among exogenous bacteria and protect them from being eliminated by their own immune system?”

In some symbioses, immune responses are modulated in a manner that permits stable association with beneficial microorganisms [2]. In *Bathymodiolus* mussels, the gills serve as direct contact sites with the external environment while also sites for accommodating symbionts. Recent studies on *Bathymodiolus japonicus* have demonstrated that the phagocytic mechanisms of the gill cells are closely linked to a series of processes, including symbiont engulfment, selection, retention, and/or digestion [22]. Furthermore, it has been shown that a central metabolic regulator, mechanistic target of rapamycin complex 1 (mTORC1), controls the subsequent fate of symbionts and exogenous bacteria—either retention or digestion—in response to presence or absence nutrients supply from bacteria including symbionts [23]. Phagocytosis is classically known as an immune response that engulfs and eliminates pathogens [24,25] and is thought to have significantly contributed to the acquisition of symbionts and the establishment of intracellular symbiosis [1,6,18,26]. From both nutritional and immunological perspectives, this relationship demands intricate coordination: the host must simultaneously tolerate symbionts that provide nutrients while activating defense responses against pathogens. The integration of nutrient sensing and immune modulation thus represents a critical interface for maintaining stable symbiosis. It is possible that similar mechanisms are evolutionarily conserved in other species of this genus, necessitating a reevaluation of the role of host defense systems in the control of symbiosis.

This review offers comprehensive insights into the host defense mechanisms that regulate the establishment and maintenance of intracellular nutritional symbiosis in *Bathymodiolus* mussels through the synthesis of existing knowledge and recent studies on *B. japonicus*. The key to unraveling the paradox of tolerating symbionts and eliminating other bacteria resides in the immune defense system and the precise molecular switching mechanism of the phagocytic process in *Bathymodiolus* mussels. Here, we discuss how the host immune system is spatially compartmentalized into hemocytes, which are responsible for systemic elimination, and gill cells, which handle local symbiotic acceptance. We also argue that the phagocytic process in gill cells is intricately regulated by a regulatory mechanism centered on mTORC1, which is coupled with nutrient supply from symbionts. This review aims to deepen our understanding of intracellular nutritional symbiosis as an adaptive strategy for deep-sea life by integrating perspectives from immune regulation, metabolic coordination, and evolutionary context.

## 2. Innate Immune Defense in *Bathymodiolus* Mussels

### 2.1. Gill Defense

**Figure 1:**
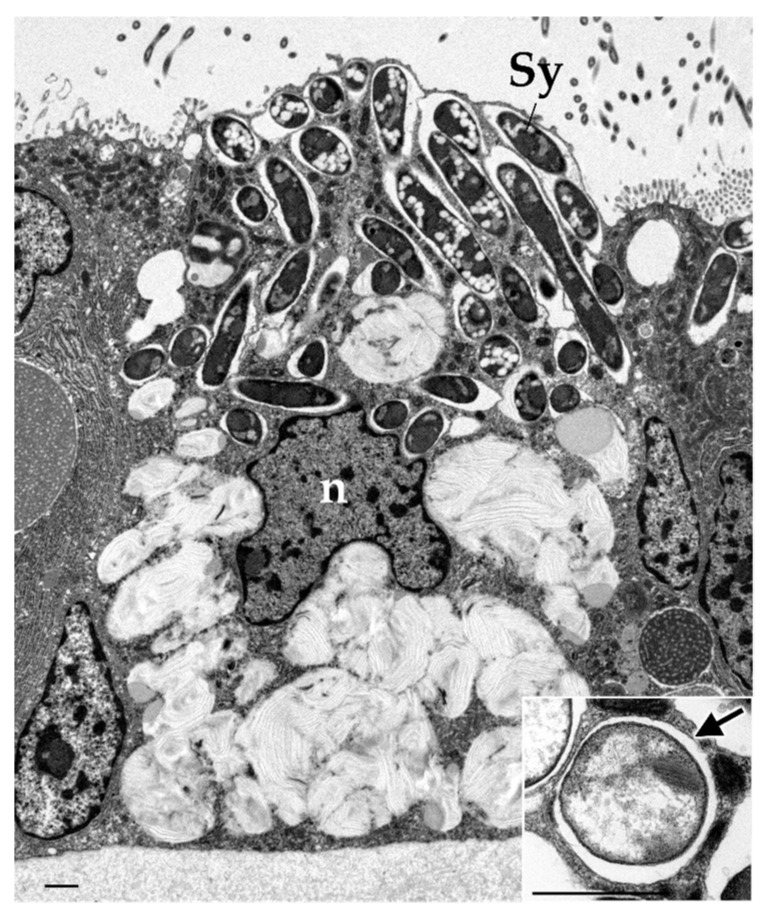
Transmission electron micrograph of the gill of *Bathymodiolus japonicus* shows a bacteriocyte harboring symbionts. Inset shows a higher-magnification micrograph of symbiosome (arrow) containing symbiont in other bacteriocyte. Experimental procedures were performed as described in Tame et al., 2023 [23]. The sections were observed using a H-7650 electron microscope (Hitachi) operated at 100 kV. n, nucleus; sy, symbiont. Scale bar, 1 μm.

The gills of *Bathymodiolus* mussels, as observed in other related bivalve species, form a lamellar structure composed of epithelial cells that are involved in respiration and the collection and transport of suspended particles [27,28]. A distinctive feature of *Bathymodiolus* mussels is the presence of intracellular symbionts such as sulfur-oxidizing and methane-oxidizing bacteria, in bacteriocytes contained in the gill filaments [6,28]. These symbionts are harbored within single-membrane-bound vacuoles referred to as symbiosomes in bacteriocytes (Figure 1). Mussels largely rely on their symbionts for nutrition, and their overall health status is known to decline if symbionts are lost during aquarium rearing in the absence of essential energy and carbon sources, such as hydrogen sulfide and methane [29,30].

In bivalves, the gill epithelium is directly exposed to environmental particles and microorganisms and therefore serves as a frontline site of the immune response [31,32]. *Bathymodiolus* mussels are no exception, and they may be exposed to the risk of infection by pathogenic exogenous microorganisms in their habitat. However, at the same time, the mussels as hosts need to maintain a long-term symbiotic relationship with symbionts, and thereby face the challenge of balancing immune defense with the regulation of symbiosis.

Similar to shallow seawater mussels, *Bathymodiolus* mussels have been shown to possess evolutionarily conserved immune defense factors, including Toll-like receptors (TLRs), NF-κB signaling pathways, antimicrobial peptides, and oxidative stress-related factors [33,34,35,36,37]. These factors play a central role in the recognition and elimination of exogenous pathogens, indicating that their immune defense systems are capable of exerting immune responses. Some immune molecules, such as TLRs, C-type lectins, peptidoglycan recognition proteins (PGRPs), and antimicrobial peptides, are predominantly expressed in the gill and mantle [35,36,37]. Notably, RNA-seq and qPCR analyses reported in previous studies consistently demonstrated that immune-related genes such as TLRs and antimicrobial peptides are expressed at higher levels in the gill than in the mantle, indicating gill tissue-specific immune activation [14,15,16]. These findings suggest that the gill plays a dual role in host defense against exogenous bacteria and in maintaining cooperation with symbionts and dynamically regulating the balance between them.

### 2.2. Hemocyte Defense

Hemocytes in *Bathymodiolus* mussels also play a central role as cellular factors in the host immune defense system. To date, in bivalve species, hemocytes have been broadly categorized into two types, including agranulocytes (hyalinocytes) and granulocytes [38,39]. Although classification criteria may vary among bivalve species, it has been shown that several *Bathymodiolus* mussels have at least three types of hemocytes: agranulocytes, basophilic granulocytes, and eosinophilic granulocytes [34,40].

Eosinophilic and basophilic granulocytes can phagocytose and digest bacteria with lysosomes, and eosinophilic granulocytes are characterized by the highest phagocytic activity [34,40]. Studies on *B. azoricus* have reported that hemocytes exhibit typical innate immune responses, such as morphological changes and superoxide production, in response to pathogen-associated molecular patterns (PAMPs), such as lipopolysaccharide (LPS) [34,41]. The expression of Rel-homology genes, antimicrobial peptides (mytilin-like), components of the mitogen-activated protein kinase (MAPK) signaling pathway, and oxidative stress-related factors have also been identified, supporting the notion that evolutionarily conserved cellular immune mechanisms mediated by hemocytes are maintained and exerted in *Bathymodiolus* mussels. These findings provide important insights into the universality of immune responses among bivalves and offer valuable clues for understanding the adaptive evolution of immune defense systems, particularly through comparative analyses of shallow seawater species.

### 2.3. Compartmentalization of Immunity and Symbiosis

**Figure 2:**
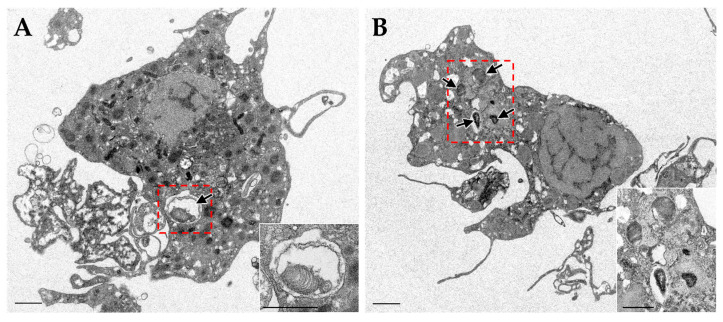
Transmission electron micrographs of hemocyte in *B. japonicus* show that (**A**) the eosinophilic granulocyte and (**B**) the basophilic granulocyte engulfed dead symbionts (arrows). Insets indicate that a higher magnification of a red dashed square in (**A**,**B**) show dead symbionts engulfed in the hemocytes. These figures are modified from the author’s doctoral dissertation (Tame, 2018). Scale bar, 1 μm [42].

In bacterial exposure experiments using fluorescence-labelled bacteria in adult *B. japonicus*, the bacteria were predominantly localized within the gill cells [23]. Rarely, the bacteria were confirmed to be present only in the hemocoel of the mantle, and these were phagocytosed by granulocytes in hemocytes. While engulfment of bacteria was observed primarily in gill cells, hemocytes may act downstream to eliminate pathogens released into the hemocoel. Hemocytes potentially exert bactericidal activity against exogenous bacteria [34,40,41], suggesting that they function as typical immune effectors, which are primarily involved in the elimination of pathogens. The granulocytes were also observed to engulf dead symbionts (Figure 2). Considering the low probability of internalization of exogenous bacteria from tissue cells other than the gills and hemocoel, phagocytosis by hemocytes is likely an elimination response rather than a selective internalization or transport of symbionts. However, their phagocytosis against symbionts may also function as a key component of a homeostatic loop for symbiont turnover, removing dead or senescent bacteria and recycling their components for the host. Taken together, these results suggest that the immune defense system of *Bathymodiolus* mussels is spatially compartmentalized into two distinct defense strategies: a systemic mechanism for sustaining homeostasis with elimination of pathogens and useless symbionts mediated by hemocytes, and a regulatory defense mechanism in the gills to facilitate the selective acceptance of symbionts. This spatial and functional division of immune roles in the living body might enable the host to effectively reconcile “defense” and “symbiosis”.

However, the involvement of hemocytes in the symbiotic system of *Bathymodiolus* mussels remains unclear. Further in vivo investigations of the functional roles of hemocytes are required to gain a deeper understanding of the control mechanisms that balance symbiosis and immunity. In the symbiotic association between the squid *Euprymna scolopes* and the luminescent bacterium *Vibrio fischeri*, the epithelial cells of the light organ take up bacteria from seawater and selectively accept *V. fischeri* through a process termed “winnowing” [43,44,45]. During this process, the establishment of symbiosis is thought to suppress the phagocytosis of symbionts by circulating macrophage-like hemocytes, suggesting that the symbiotic system is maintained through host immune tolerance. Thus, in *Bathymodiolus* mussels, it is necessary to consider the possibility that the symbiont selection mechanism in local tissues and the broad-ranging response of the systemic immune system cooperatively maintain a balance between symbiosis and defense. An in vivo analysis of their response characteristics to symbionts and the underlying immune regulatory mechanisms is expected to provide a fundamental basis for understanding immune defense and symbiotic interactions.

## 3. Gill Epithelial Cells as a Mediator of Symbiosis

### 3.1. Horizontal Acquisition and Developmental Distribution

*Bathymodiolus* mussels are known to acquire symbionts from the symbiont acquisition via horizontal transmission based on ultrastructural observations of endocytosis-like uptake structures directed toward symbionts and phylogenetic incongruence between the host and symbiont [17,18,19]. Further support for horizontal transmission has been provided by the observation that symbionts can be reacquired after being lost under laboratory conditions [30] and by the absence of symbionts in the early developmental stages [19,46,47].

Mussel larvae in the early pediveliger stage are in an aposymbiotic state, completely lacking symbionts, with no symbionts detected in any tissue, including the gills [47]. In the post-metamorphic plantigrade stage, symbionts are first observed on the outer surface and intercellular spaces of the gills, followed by the initiation of colonization within the gill cells [19,46,47]. During this stage, symbionts also transiently localize to tissues other than the gills, such as the mantle, foot, and retractor muscles, suggesting that multiple tissues may be conducive to symbiont colonization in the early phase of acquisition [19,46]. Eventually, as gill development progresses, the localization of symbionts becomes restricted to the gill cells, and they are no longer detected in other tissues [47]. This spatial shift in symbiont distribution is thought to reflect the onset of host immune maturation and selective acceptance mechanisms, accompanied by gill development [19]. The spatial compartmentalization in local acceptance by gill and systematic elimination by hemocytes is not fixed at the larval stage but is developmentally acquired and reinforced as the immune system matures and gill specialization progresses. This developmental shift highlights the dynamic nature of host–symbiont interactions and suggests a close link between immune maturation and symbiont localization.

Transcriptome analysis during the developmental stages from larvae in *Gigantidas platifrons* has revealed an upregulation of pattern recognition receptors (PRRs: TLRs, C-type lectins) and lysosome-related genes in the gills during the process leading to adulthood, whereas their expression was downregulated during the larval stage, suggesting a correlation with multi-tissue localization in the early phase [48]. Immune-related factors such as PGRPs, TLRs, and bactericidal permeability-increasing proteins (BPIs) are highly expressed in the gill tissues of adult mussels [15]. In addition, the localization of TLR2 and leucine-rich repeat protein 1 (LRR-1) to gill cells has also been demonstrated by in situ hybridization analysis. Furthermore, in bacterial exposure experiments on adult mussels, the majority of the exposed bacteria were observed to localize intracellularly or on the surface of gill cells [22]. As mussels develop into adults, the acquisition and colonization of symbionts become progressively restricted to the gill epithelial cells, and the maturation of the immune response is considered to be involved in the spatial control of symbiont localization. The gills in mussels serve not only as the primary organs in direct contact with the external environment but also as a respiratory organ capable of efficiently acquiring inorganic reductants such as oxygen, hydrogen sulfide, and methane, thereby providing an ideal environment for nutritional symbiosis [6,19]. Thus, the specialization of the gill tissue as the site of symbiosis is physiologically and immunologically reasonable. This spatial shift in symbiont distribution may reflect a co-evolutionary trajectory in which the physiological specialization of the gills for efficient particle acquisition and respiration develops in concert with the maturation of host immune responses, such as the upregulation of PRRs. This dual specialization creates a highly permissive and selective niche that is ideal for symbiosis. Although the detailed host immune mechanisms that spatially control symbiont localization are not yet fully understood, this remains an important subject that is expected to be clarified in future research, contributing to the understanding of functional differentiation in tissue cells and the mechanisms of immune system development and regulation.

### 3.2. Disappearance and Reacquisition of Symbionts

The symbiotic system of adult *Bathymodiolus* mussels exhibits a high degree of plasticity, allowing both the loss and reacquisition of symbionts. When mussels are reared in sterile filtered seawater without the addition of energy sources for symbionts, such as hydrogen sulfide or methane, the symbionts gradually disappear from the gill cells within approximately one to two months [23,30,49]. In contrast, when mussels that have lost their symbionts are either returned to their natural habitat or reintroduced into an aquarium supplemented with hydrogen sulfide or methane, the symbionts are re-harbored in the gill cells within a period of several days to weeks [19,30]. This phenomenon is considered to reflect the reacquisition of symbionts present in the natural environments or aquarium water.

The presence of symbionts within the gill cells of mussels influences host immune control mechanisms. Several PRRs, immune-related genes, and apoptosis-related factors have been upregulated in mussels with lost symbionts [50], suggesting that host immune defense is activated with loss of symbionts and intracellular homeostatic disruption. Autophagy-related genes and components of the ubiquitin-proteasome system were also upregulated, implying the activation of compensatory pathways for the removal and recycling of unnecessary cellular structures and proteins [51]. Indeed, in *B. japonicus*, it has been demonstrated that the phagosome digestion of symbionts is promoted through mTORC1 under methane-depleted conditions, leading to the degradation and utilization of symbionts as nutritional resources [23] (see also Section 4.3 for details). Although it is known that mTORC1 activates the proteasome system and suppresses autophagy [52], phagosome digestion of symbionts occurs independently at the individual cell level, suggesting that the activation of these degradation systems may vary among gill cells [23]. In contrast, in mussels re-exposed to symbionts, excessive immune responses and intracellular degradation pathway activities tended to be suppressed, suggesting that the presence of symbionts induces the reorganization of the molecular mechanisms underlying immune regulation in adult mussels [51]. The activation of immune-related factors following the loss of symbionts may be linked to the facilitation of their reacquisition. It may be more appropriate to consider this compensatory response for homeostatic maintenance in a symbiont-dependent manner rather than a response to immune tolerance.

### 3.3. Selective Specificity of Symbionts

Symbionts maintained by each host exhibit a certain degree of host species specificity. For instance, *B. azoricus* harbors both sulfur- and methane-oxidizing bacteria, whereas *B. thermophilus* and *B. septemdierum* contain only sulfur-oxidizing bacteria, and *B. japonicus* and *G. platifrons* harbor methane-oxidizing bacteria [6,28,53,54]. Even in habitats where multiple *Bathymodiolus* species coexist, such as *B. japonicus* and *G. platifrons* off Hatsushima in Sagami Bay, it has been reported that each host maintains symbionts of different lineages [6,12,55]. This host-symbiont specificity has also been observed in the early developmental stages of larvae [19,46,47]. However, this specificity is not absolute, as similar symbionts have been detected among closely related host species [28,53]. In addition, cases have been confirmed where the clades of symbionts maintained within the same species differ owing to geographical and environmental factors [12,20,56].

Despite the widespread presence of bacteria, including symbionts in the environment, the clade of symbionts maintained by each host individual is consistent and typically dominated by a single lineage, suggesting the establishment of “monocladal selective specificity,” in which a particular symbiont clade is preferentially maintained. For example, metagenomic analyses of the symbionts associated with *B. brooksi* have shown that despite the symbionts being environmentally derived, they form genetically distinct clades in each individual mussel and are maintained within the host via self-infection after acquisition [20,21]. In *B. septemdierum*, although metabolically distinct symbionts have been found to coexist within a single individual, a single clade is predominant in each gill cell [57]. These findings indicate that, even if multiple symbiont clades exist in a population of the same host species, the coexistence of different symbiont clades within an individual host is rare, and the symbiont composition of each individual tends to be highly consistent. Therefore, the symbiotic relationship in *Bathymodiolus* mussels is appropriately regarded as a “selective specificity” that combines species specificity and flexibility, while being based on horizontal transmission. Collectively, these findings indicate that host mussels possess mechanisms that selectively retain beneficial symbionts. It is considered that the evolutionary stability of their symbiotic system is contributed to by the establishment of unique symbiotic relationships in each mussel through such selective mechanisms, along with the flexibility to enable environmental adaptation and symbiont re-acquisition.

## 4. Intracellular Nutritional Symbiotic Mechanism Through Phagocytosis

### 4.1. Phagocytosis for Symbiont Acquisition

Phagocytosis in eukaryotic cells is a widely recognized biological defense mechanism that engulfs, digests, and eliminates exogenous bacteria [24,25]. This is considered a critical step in establishing intracellular symbiosis with bacteria [1,6,18,26]. Exposure experiments using fluorescently labelled exogenous bacteria in *B. japonicus* demonstrated that gill cells possess the ability to phagocytose bacteria [22]. In pathogens, the secretion systems (Type III and Type IV secretion systems) have been implicated in host cell invasion, immune evasion, and colonization control, and similar mechanisms have been reported in symbiotic microorganisms [58,59,60]. However, no genes encoding such secretion systems have been found in the symbionts of *Bathymodiolus* mussels [14,16,23,61,62], suggesting that these symbionts lack the molecular machinery for direct host invasion or immune evasion. Even in experiments using excised gill filaments, phagocytic responses against bacteria by gill cells were observed [22], suggesting that active phagocytosis by gill cells contributes to symbiont acquisition in adult mussels.

In contrast, phagocytosis in mussels occurs indiscriminately regardless of the bacterial species [22]. Although PRR expression has been reported in the gill cells of *Bathymodiolus* mussels [15,50], the discrimination between symbiotic and non-symbiotic bacteria may be limited prior to internalization. Phagocytic and immune-related activities of gill cells have also been reported in non-symbiotic mussels. Gill cells were shown to internalize microplastic particles [63], and transcriptomic analyses further demonstrated their active role in the immune response to bacterial challenge [64], while community-level studies revealed selective associations with the environmental microbiota in related mussel species [65]. These reports suggest that phagocytosis and internalization in the gill cells may be not unique to symbiotic mussels. While *B. japonicus* also exhibits specific selectivity towards its symbionts [6,12,55], the non-selective engulfment of bacteria in gill cells may increase opportunities to acquire beneficial symbionts, and thus contribute to the “flexibility” of *Bathymodiolus* mussels to establish and maintain unique symbiosis in each of them.

The acquisition of symbionts through non-selective phagocytosis has also been documented in the intracellular symbiosis between cnidarians and symbiotic algae (*Symbiodinium*) [26]. Symbiotic algae are internalized by host gastrodermal cells via phagocytosis and are maintained within symbiosomes. Although phagocytosis is also non-selective, the mechanism by which only symbiotic algae are maintained has been suggested to involve post-phagocytic processes [66]. In *B. japonicus*, the acquisition of symbionts is not accomplished merely through surface attachment or passive diffusion; active phagocytosis by the host serves as the starting point. The establishment and maintenance of intracellular symbiosis is subsequently achieved through the suppression of digestion within phagosomes [23] (see also Section 4.3 for details). These observations indicate that the symbionts may not escape elimination but are selected through control of the phagocytic process, suggesting that the phagocytic mechanism plays an essential role in the establishment of intracellular symbiosis.

### 4.2. Phagosome-Derived Symbiosome

Generally, during the phagocytic process, as a host immune defense mechanism, immature phagosomes containing engulfed bacteria undergo a series of stepwise and coordinated maturation events, ultimately forming phagolysosomes. Specific factors are involved at each stage of the process [24,67,68]. Immunohistochemical analysis using *B. japonicus*-specific antibodies revealed that Ras-related protein Rab-9 (Rab9), lysosomal-associated membrane protein 1 (LAMP1), vacuole-type H+ adenosine triphosphatase (V-ATPase), and mannose-6-phosphate receptor (M6PR), which are key molecules involved in the maturation of late phagosomes to phagolysosomes, were localized on phagosomes containing fluorescent labelled exogenous bacteria [23]. Rab9 plays a role in delivering lysosomal enzymes from the trans-Golgi network to the phagosome via M6PR, and the delivered enzymes are activated within phagosomes acidified by V-ATPase [24,67,68]. LAMP1 serves as a marker for fusion with lysosomes and simultaneously participates in stabilizing the membrane structure after fusion. Indeed, acidification and esterase activity were confirmed within phagosomes containing these bacteria, indicating that phagocytosed exogenous bacteria were rapidly digested within the phagosomes for elimination [22]. Although the clear presence of lysosomes has not been confirmed within gill cells [23], LAMP1 is thought to be involved in the fusion of phagosomes and vesicles containing lysosomal enzymes delivered via vesicular transport.

Notably, the same sets of immunohistological markers are also localized on symbiosomes harboring symbionts [23]. This finding indicates that symbiosomes are derived from phagosomes and/or phagolysosomes and that their structural and molecular frameworks are conserved even in symbiotic relationships. Thus, the symbiotic system in *Bathymodiolus* appears to utilize the phagosome digestion mechanism in the host’s immune defense system while achieving a symbiotic state through functional control of this pathway. However, a striking contrast is observed from a functional perspective. While phagosomes containing exogenous bacteria rapidly undergo acidification and exhibit esterase activity leading to digestion, these digestive processes are suppressed within symbiosomes, allowing the symbionts to be stably maintained without degradation [22,23]. The difference in digestive processing between these symbionts and exogenous bacteria suggests that the processes in these two compartments are independently regulated. Such a state—structurally phagosome yet functionally non-digestive—has also been shown in the symbiosis between cnidarians and *symbiodinium* [26], implying that the repurposing of phagosomes may be one of potentially universal evolutionary strategy for intracellular symbiosis in the animal kingdom [1].

### 4.3. Integrated Regulation Between Phagocytosis and Nutrient Signaling by mTORC1

**Figure 3:**
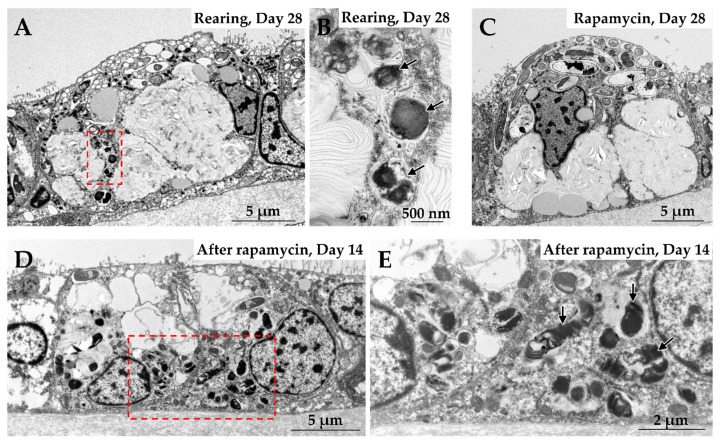
Transmission electron micrographs show bacteriocytes of *Bathymodiolus japonicus* in rearing experiments. (**A**) Mussels were reared in a methane-depleted aquarium for 28 days. (**B**) A higher magnification of a red dashed square in (**A**) shows digesting symbionts. (**C**) Mussels were reared in the aquarium with rapamycin for 28 days. The symbionts were retained in bacteriocytes. (**D**) Mussels were reared in the aquarium without rapamycin for 14 days, after being reared with rapamycin for 14 days. (**E**) Higher magnification of a red dashed square in (**D**) shows digestion of the symbionts, which were retained during rearing with rapamycin. Experimental procedures were performed as described in Tame et al., 2023 [23]. The sections were observed using a H-7650 electron microscope (Hitachi, Tokyo, Japan) operated at 100 kV.

The specific regulatory function of mTORC1 in *B. japonicus* is clearly demonstrated by the cellular response observed when the nutrient supply from the symbionts is disrupted [23]. When the mussels are reared under methane-depleted conditions, which deprive the symbionts of their primary energy source, esterase activity becomes detectable within the symbiosomes within a few weeks, accompanied by a reduction in the number of symbionts. When rapamycin, an inhibitor of mTORC1 kinase activity, was added to the mussel rearing experiments, esterase enzymes were not detected, and symbionts retained in the symbiosomes even after 28 days of rearing (Figure 3). This phenomenon was also observed in bacterial exposure experiments with *B. japonicus*. When rapamycin was added to the experiments, esterase activity was not detected in phagosomes containing exogenous bacteria, and phagosome digestion was suppressed.

**Figure 4:**
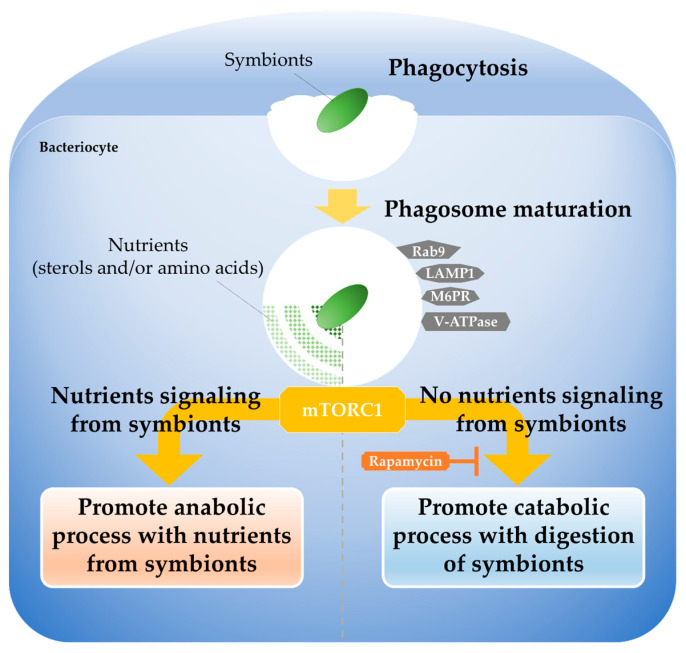
Schematic model adapted and modified from Tame et al. (2023) [23]. Host mussels acquire symbionts by gill cell phagocytosis, and then select retaining or digesting them during phagosome maturation process. mTORC1 receives nutrient signaling from the symbionts promote anabolic metabolism utilizing nutrients derived from them. In contrast, when nutrient supply from symbionts reduces, or when other bacteria do not provide any nutrients to hosts, mTORC1 promotes catabolic metabolism, leading to the digestion of these bacteria. This mTORC1-mediated phagosome digestion is inhibited by rapamycin.

Given that phagosome digestion of both symbionts and exogenous bacteria was suppressed by rapamycin treatment, it is suggested that both are subjected to mTORC1 mediated selection via the same phagocytic pathway. Although symbionts lack molecular factors such as secretion systems to evade phagosome digestion, previous studies in *Bathymodiolus* mussels have reported that the symbionts supply the host mussels with methane-derived carbon compounds, such as amino acids, sterols, and their intermediates [14,15,16,23,62]. Additionally, mTORC1 is known to integrally coordinate anabolic processes such as protein and cholesterol biosynthesis and catabolic processes such as protein degradation in response to an intracellular supply of nutrients such as amino acids and lipids [52,69,70,71]. These findings suggest that mTORC1 promotes the phagosome digestion of symbionts with reduced nutrient supply and for exogenous bacteria that do not provide nutrients. When the nutrient supply from the symbionts is sufficient, mTORC1 in host prioritizes anabolic pathways; however, when this nutrient supply decreases, it functions as a metabolic switch that promotes catabolic processes (phagosome digestion) to actively degrade and utilize the symbionts as an alternative nutrient source (Figure 4). This dynamic regulation by mTORC1—which switches between “maintenance” and “degradation/utilization”—is thought to reflect the host’s metabolic flexibility and adaptive strategy to the unstable environments of deep-sea hydrothermal vents and cold seeps, where the supply of chemoautotrophic nutrients fluctuates.

In mammalian studies, it is known that when intracellular nutrients such as amino acids and lipids are abundant, mTORC1 becomes activated on the lysosomal membrane, which in turn promotes anabolic processes like protein and lipid synthesis [52,69,70,71]. Conversely, the depletion of intracellular nutrients induces the inactivation and dissociation of mTORC1 from the lysosomal membrane, thereby promoting catabolic processes such as autophagy, which enables the cell to recycle internal components for survival. In *B. japonicus*, mTORC1 is localized on both symbiosomes harboring symbionts and phagosomes containing exogenous bacteria [23], indicating that mTORC1 is present in an active state in both vacuoles. When rapamycin was applied the experiments, inhibition of mTORC1 activity did not induce autophagy-like responses (Figure 3). Although the underlying molecular mechanisms remain unclear, it is possible that mTORC1 functions dependently on the symbiont’s nutrient supply status and intrinsically possesses a regulatory switch function in itself. A key focus of future research will be to elucidate how mTORC1 senses the nutrient signals derived from the symbionts to determine the fate of symbiosomes and phagosomes. It is hypothesized that nutrients supplied by the symbionts signal to mTORC1 via nutrient sensors located on the lysosomal membrane (e.g., Rag GTPases) or specific intracellular transporters that are essential for mTORC1 activation [52,69,70,71]. For instance, host transporters responsible for specific nutrients such as amino acids or lipids may sense the intracellular concentrations of these symbiont-derived metabolites and relay this information to mTORC1, thereby determining the fate of the symbiosome (phagosome) according to the cell’s metabolic balance. Thus, mTORC1 serves as a molecular hub integrating crosstalk between the host’s immune defense system (phagosome digestion) and nutrient metabolism, enabling deep-sea mussels to decide whether to maintain their internal microbes as “beneficial symbionts” or digest them as “foreign entities for utilization.”

## 5. Discussion and Future Perspectives

The symbiosis regulation model proposed in this review suggests that *Bathymodiolus* mussels achieve active selective retention by coupling the immune pathway of phagocytosis with metabolic control centered on mTORC1. This supports the notion that the host immune system functions not merely as a pathogen elimination mechanism but is also repurposed as a symbiotic interface. This strategy is fundamentally different from the passive immune tolerance observed in the squid–*Vibrio* symbiosis, where symbionts are protected through suppression of the host’s systemic immune responses [43,44,45]. In *Bathymodiolus* mussels, the immune response is not suppressed but is dynamically regulated according to the nutritional state. Hemocytes are responsible for systemic pathogen clearance, whereas gill epithelial cells mediate localized symbiont acceptance, establishing a spatial compartmentalization of immune function. In the gill cells, mTORC1 modulates phagosome digestion in response to nutrient supply, thereby balancing the maintenance and elimination of symbionts.

The role of mTORC1 in stabilizing the symbiotic niche likely differ substantially depending on the host species and the nutritional modality of the symbiosis, which represents a key point of divergence between the present study and recent research in cnidarians [66]. In the symbiosis between cnidarians and *Symbiodinium*, photosynthetic products supplied by symbiotic algae have been suggested to activate mTORC1, which, in turn, contributes to the stabilization of the intracellular niche (symbiosome) harboring symbiotic algae in coordination with host metabolism. A notable difference in *B. japonicus*, however, is that the inhibition of mTORC1 kinase activity in this system leads to the loss of symbiotic algae and the breakdown of the symbiotic relationship. This contrasting outcome might indicate that mTORC1 plays multifaceted roles depending on the species, modality of symbiosis, and phase of symbiosis, whether during establishment or maintenance. In *B. japonicus*, it is conceivable that mTORC1 regulates metabolic activities by sensing nutrient signals supplied from symbionts, thereby playing a role in switching between symbiont retention and degradation. That is, while symbiosis is maintained as long as the nutrient supply from the symbionts is sustained, the symbionts become a target for degradation when the supply is cut off. In cnidarians and *symbiodinium*, mTORC1 is thought to contribute to the establishment of a cellular environment that enables the long-term and stable maintenance of symbiotic algae by sensing and integrating the nutrient supply from them. This functional divergence suggests that, although the mTORC1 signaling pathway is highly conserved across metazoans [72,73,74,75], its downstream effectors and interacting partners have been evolutionarily rewired to meet the specific ecological and physiological demands faced by the host, such as environmental variability in the deep-sea and the need for nutrient recycling.

The current findings show seemingly distinct regulatory modes in terms of conditional maintenance in mussels and constitutive stabilization in cnidarians. However, both share a common basis for the control of mTORC1 activity in response to nutrient signals from symbionts and may represent continuous plasticity. Studies on intracellular pathogens have shown that mTORC1 is involved in bacterial clearance by promoting phagosome maturation. Conversely, certain pathogens manipulate the mTORC1 pathway to inhibit phagosome maturation and utilize it as a survival strategy within the host cells [76,77]. Thus, symbionts and pathogens may utilize host membrane trafficking and signaling pathways, potentially altering the host’s decision between acceptance and elimination. Collectively, mTORC1 functions as a central regulatory factor that integrates the crosstalk between the phagocytic pathway and nutrient signaling. Future studies focusing on the identification of mTORC1 downstream effectors and the dynamics of nutrient exchange between host and symbionts are essential to reveal the full complexity of this symbiotic regulatory system.

In systems where symbionts are acquired via horizontal transmission, stringent selection mechanisms are essential to ensure the specificity and stability of the symbiosis [1,2,28,75]. In *Bathymodiolus* mussels, the selectivity is established through a post-phagocytic process in which phagosome fate is determined via mTORC1 following the non-selective phagocytosis of bacteria by the gill cells. This nutrient signal–based regulation also suggests that the host may be indirectly involved in mediating competition among environmental microbes [75,78]. mTORC1 may sense signals from the symbionts that provide nutrients most efficiently and maintain their niche, while actively eliminating bacterial strains considered inefficient or foreign, thereby contributing to quality control and the maintenance of clonal purity within the symbiosis. This dynamic metabolic regulation is distinct from simple immune tolerance. Some theoretical studies have suggested that tolerance toward defensive symbionts may entail an evolutionary cost over the long term by reducing the host population’s capacity to eliminate pathogens [79]. The mTORC1 model in *Bathymodiolus* mussels is considered a strategy that avoids this potential evolutionary cost by dynamically activating or suppressing the defense system based on metabolic necessity, rather than relying on simple tolerance. This may be attributable to the unique functional differentiation of the gill cells. Recent single-cell analyses have generated a gill cell map for deep-sea mussels, revealing that the gill bacteriocytes are highly coordinated with symbionts in the metabolism of sterols, carbohydrates, ammonia, and other compounds to support symbiont cultivation [80]. This suggests that they constitute a metabolic network with the nutrient signals through mTORC1. Considering that the expression of PRRs and lysosome-related genes is tightly regulated according to developmental stage and symbiotic state [15,48], the gill cells of *Bathymodiolus* mussels, particularly bacteriocytes, are distinguished from conventional immune cells (hemocytes) and regarded as specialized immunometabolic cells that integrate immune responses (phagocytosis) with nutrient assimilation. The identification of the mTORC1 switch mechanism provides strong support for establishing *Bathymodiolus* mussels as a “deep-sea immune-metabolic model,” in which nutrient sensors regulate immune functions, and greatly enhances its value for comparative studies on the universality and diversity of symbiosis control across metazoans.

Recent advances in the analysis of immune and metabolic regulatory factors have revealed conserved cellular mechanisms that mediate host–symbiont interactions in mussels. Accumulating evidence strongly suggests that intracellular nutritional symbiosis in mussels is supported by a finely tuned adjustment of the host immune defense system, which originally functioned to eliminate foreign bacteria. At the heart of this control is the central metabolic regulator, mTORC1. A series of immune-and mTOR-related factors are evolutionarily conserved across the genus *Bathymodiolus*. Although symbiotic strategies exhibit species-specific flexibility and selectivity, metabolic interactions with symbionts have been consistently confirmed in mussels. Thus, mTORC1, as an integrated regulator of the host immune defense and nutrient metabolic systems, may also be involved in other *Bathymodiolus* mussels.

As presented in this review, in *B. japonicus*, mTORC1-dependent control of phagosome digestion functions as a crucial regulatory mechanism that determines the fate of internalized bacteria, choosing whether to maintain beneficial symbionts or eliminate them as unwanted bacteria. This switch between “maintenance” and “digestion” appears to confer mTORC1 with intent, but it is more likely to be a metabolically driven molecular switching mechanism that responds to nutritional signals from the symbionts. Interestingly, rearing experiments under methane-depletion conditions showed significant changes in the intracellular dynamics of symbiont retention and digestion, although the mRNA expression levels of phagocytosis- and mTOR-related genes did not show any major changes. This highlights the importance of future analyses of functional states, protein levels, and spatial localization of these factors from a morphological perspective.

Despite the lack of an adaptive immune system, the fact that *Bathymodiolus* mussels utilize evolutionarily conserved innate immune pathways to recognize and accommodate symbiotic bacteria, thereby establishing a highly specific and stable intracellular symbiotic relationship, raises a fundamental question in evolutionary immunology. Can the immune system inherently function as a control interface for symbiosis?

To validate the “integrated model of symbiosis regulation” centered on mTORC1 proposed in this review, we present the following future research topics as a roadmap.

1. Deciphering the molecular basis of the immune–symbiosis interface: To understand how symbiotic bacteria are distinguished from foreign bacteria that should be eliminated, it is essential to identify key signaling molecules involved in the nutritional signaling pathways that control the transition from bacterial elimination to symbiont maintenance, and to pinpoint their intracellular localization.
2. Elucidating bidirectional metabolic signals between symbionts and mTORC1: To gain a deeper understanding of the nutritional control network in symbiosis, it is necessary to clarify how symbiont-derived metabolites (e.g., amino acids, lipids, and sterols) affect host mTORC1 activity and downstream phagosome digestion processes, using methods such as metabolic inhibition experiments.
3. Advancing symbiosis research from a comparative immunology and evolutionary perspective: Functional comparisons of symbiosis-related immune and metabolic regulatory factors across various host–symbiont systems will provide insights into the evolutionary conservation and plasticity of these networks. Furthermore, evolutionary tracing of the acquisition and modification processes of symbiosis-related factors is extremely important for understanding the universality and diversity of symbiosis.
4. Exploring the minimal molecular basis for symbiont acquisition in non-symbiotic close relatives: Exploring symbiosis-related genes in non-symbiotic close relatives will be useful for identifying the minimal genetic components required for the establishment of symbiosis. This knowledge may potentially lead to the reconstruction of artificial symbiotic systems and their applications in various fields of biology.

Ultimately, validation of an integrated model centered on mTORC1 that unifies immune control, nutritional sensing, and symbiosis control is expected to lead to a deeper understanding of the principles that govern the boundary between immune defense and symbiotic tolerance. This principle is likely fundamental for enabling host–microbe coexistence in diverse taxonomic groups.

## Data Availability

No new data were created or analyzed in this study. Data sharing is not applicable to this study.
